# Acceptability of oral health care: a scoping review of reviews to inform framework development

**DOI:** 10.1186/s12903-026-09124-6

**Published:** 2026-07-31

**Authors:** Sonica Singhal, Shelly Dhaliwal

**Affiliations:** 1 Public Health Ontario, Toronto, ON Canada; 2 Faculty of Dentistry, University of Toronto, Toronto, ON Canada; 3 Division of Epidemiology, Dalla Lana School of Public Health, University of Toronto, Toronto, ON Canada

**Keywords:** Acceptability, Access to Care, Barriers and Facilitators, Dental Care [MeSH], Health Policy [MeSH], Health Services Accessibility [MeSH], Oral Health [MeSH], Patient Acceptance of Health Care [MeSH]

## Abstract

**Background:**

Canada launched the Canadian Dental Care Plan (CDCP) in 2023 to improve access by reducing cost barriers. Data from September 2025 indicate that only about half of enrollees had accessed services, highlighting that addressing affordability alone may not be sufficient to facilitate uptake. Among the multiple dimensions of access, acceptability has been the least explored in oral health, despite its influence on whether patients seek care and providers deliver it. This review synthesized acceptability-related barriers and facilitators from patient and provider perspectives to assess their influence on access to and utilization of oral health care.

**Methods:**

A scoping review of reviews was conducted. Four databases (MEDLINE, Embase, Scopus, PsycINFO) and targeted grey literature from oral health and governmental organizations were searched for English or French language sources explicitly addressing acceptability or proxy concepts in oral health. Extracted findings were analyzed thematically using an abductive approach within a constructivist/interpretivist paradigm.

**Results:**

Sixty-four sources (47 peer-reviewed reviews, 17 grey literature) met inclusion. Key patient-level barriers included anxiety, fear, cultural beliefs, and provider attributes; facilitators included behavioural or psychological support, provider's cultural competency, and shared decision making. Key provider-level barriers centered on inadequate reimbursement and gaps in clinical preparedness; facilitators included structural support and targeted training. Identified themes informed the development of the acceptability of oral health care framework.

**Conclusions:**

Acceptability is context-dependent and influenced by both patient and provider perspectives. Applying this lens can bridge gaps between coverage, reach, and utilization by ensuring that care is not only about being affordable and available, but also aligned with patient and provider needs and expectations. Integrating acceptability into oral health care policy and program planning is critical to maximizing uptake and equity.

**Supplementary Information:**

The online version contains supplementary material available at https://doi.org/10.1186/s12903-026-09124-6.

**Table:** 

Text box 1. Contributions to literature
• Implementation science has paid little attention to the concept of acceptability in oral health care, despite persistent challenges in how care is delivered and experienced.
• Evidence across oral health research indicates that acceptability shapes multiple aspects of care delivery and experience, and operates differently for varying patients and providers.
• By bringing this evidence together, this review of reviews addresses an important gap by supporting more systematic consideration of the acceptability dimension when planning and delivering oral health care.

## Background

Globally, oral diseases affect an estimated 3.7 billion people, making them among the most prevalent health conditions worldwide [1]. Despite this burden, access to oral health care remains inequitable across and within countries, with significant disparities linked to income, geography, insurance coverage, and the social determinants of health [1]. Even in countries with well-established dental care systems, utilization often falls short of need. In Canada, unlike general medical care, dental care remains largely excluded from universal health insurance, leaving most people reliant on employer-sponsored benefits, private plans, or out-of-pocket payments [2, 3]. In 2023, the federal government launched the Canadian Dental Care Plan (CDCP) to reduce cost barriers and improve access to oral health care for low- and middle-income Canadians [4]. By September 30th 2025, 5,509,911 clients had registered, yet only 3,053,502 (55%) had accessed services, suggesting that reducing financial barriers alone does not facilitate access or utilization [5].

Access to care is widely understood as a multi-dimensional construct. The framework by Penchansky and Thomas (1981), later expanded by Saurman (2016), defines six interrelated dimensions of access: availability, accessibility, accommodation, affordability, acceptability, and awareness [6, 7]. Levesque et al. (2013) further reconceptualized access as a dynamic interface between the health system and populations, shaped by system factors (approachability, acceptability, availability, affordability, appropriateness) and user abilities (to perceive, seek, reach, pay for and engage with care) [8]. While these models include acceptability, it remains one of the most ambiguous and least explored dimensions, especially in oral health care.

In broader health care, Sekhon et al.’s Theoretical Framework of Acceptability (TFA) identifies components such as affective attitude, burden, ethicality, intervention coherence, opportunity costs, perceived effectiveness, and self-efficacy, while Bucyibaruta et al. define healthcare acceptability as “a multi-construct concept describing the nonlinear cumulative combination in parts or in whole of experienced or anticipated specific healthcare from the relevant patients/participants, communities, providers/researchers or healthcare systems’ managers and policy makers’ perspectives in a given context” [9, 10]. Oral health care presents distinct acceptability challenges including higher levels of fear and stigma, frequent perception of low value, and structural separation from general health services [9, 11].

Some studies have explored how acceptability functions as a barrier or facilitator to oral health care; however, these investigations have typically examined only one or a few attributes of acceptability [12–16]. To the best of our knowledge, no review of reviews has examined acceptability as a dimension of access to oral health care (based on a search conducted on June 9, 2025). To improve utilization, a more comprehensive understanding of how oral health services are perceived and experienced is needed. This scoping review of reviews therefore asks: *How does “acceptability” as an attribute influence access to oral health care from both patient and provider perspectives?* By mapping key acceptability-related barriers and facilitators faced by patients and providers, this review aims to inform a framework that can guide the design of oral health programs that address not only affordability and availability barriers, but also the acceptability of services for the communities they intend to serve.

## Methods

### Search strategy and information sources

A scoping review of reviews was conducted following the methodological framework outlined by Arksey and O’Malley (2005) and reported according to the Preferred Reporting Items for Systematic reviews and Meta-Analyses extension for Scoping Reviews (PRISMA-ScR) guidance [17, 18] (Additional File 1). No formal protocol was registered or published for this review. Searches were designed by Public Health Ontario Library Services to capture both peer-reviewed and grey literature. Searches were limited to English or French (official languages of Canada) literature, with no date restrictions. Scientific database searches for reviews were conducted on June 9, 2025, using the following platforms: MEDLINE (Ovid), Embase (Ovid), Scopus (Elsevier), PsycINFO (EBSCO). Grey literature was identified through a combination of general web searches and three custom Google search engines targeting dental organizations (e.g., Canadian Dental Association, American Dental Association, FDI (World Dental Federation), Canadian health departments (e.g., Statistics Canada, Canadian Institute for Health Information), and international public health bodies (e.g., World Health Organization, Australian Institute of Health and Welfare, Public Health England)) on June 24 and July 2, 2025.

The strategy combined controlled vocabulary and keywords for oral health with terms related to acceptability, barriers, facilitators, and service utilization. The full MEDLINE strategy is provided in Additional File 2; strategies for other databases are available upon request. The MEDLINE strategy was adapted for web environments, accommodating search engine limitations while retaining conceptual coverage. To balance sensitivity and feasibility, up to 100 top-ranked results were screened per query from custom search engines, and the top 10 results per query were reviewed from general web searches.

### Study selection

Screening was conducted in Covidence in two stages: title/abstract review followed by full-text review. Inclusion and exclusion criteria are summarized in Table 1. To ensure reliability, a second reviewer independently screened 20% of records at Level 1 and 10% at Level 2. An agreement rate of 80% was achieved, and discrepancies were resolved through discussion between the two reviewers until consensus was reached, with adjustments applied to the remaining records accordingly. Data extraction was reviewed by the second reviewer. Grey literature sources, many of which lacked abstracts, were reviewed in full text at a single stage. Webpages were not de-duplicated due to site structure and versioning variability.

**Table 1:** Inclusion and exclusion criteria

Inclusion	Exclusion
Literature related to oral or dental health.	Literature not related to oral or dental health.
Literature that explicitly examines acceptability or includes proxy factors for acceptability such as: • Social, emotional, psychological or cultural factors. • Barriers, interventions, facilitators. • Attitudes, values, beliefs, perceptions, behaviours, preferences, motivations, safety, satisfaction, relationships.	Literature that does not explore acceptability or any proxy factors.
English or French.	Any language other than English or French.
Grey literature and reviews with clear authorship, issuing body, and methodology.	Primary studies.

### Data extraction and analysis

Relevant findings from the included reviews were extracted into Excel and qualitatively synthesized. Table 2 presents the complete set of barriers and facilitators charted across the included reviews, serving as a comprehensive record of extracted findings. The narrative synthesis in the Results is organized around the domains of the resulting acceptability framework, illustrating each theme with representative examples. Where barriers or facilitators were not explicitly labeled, they were interpreted from the findings when sufficient detail was available. 

**Table 2:** Summary of patient and provider reported barriers and facilitators of acceptability in oral health care

Author/ Organization (Year)	Title	Country of Author/ Organization	Specific Context(s)	Patient		Provider	
				Barrier	Facilitator	Barrier	Facilitator
Alzahrani AH, & Gibson J. (2018) [19]	Scoping review of the role of shared decision making in dental implant consultations	United Kingdom & Saudi Arabia	Dental Implant Consultations	Anxiety; Fear; Pain; Procedural invasiveness and risk; Financial values/perceived selfishness; Paternalism	Shared decision-making and decision-independence; Desire for restored function and aesthetics; Patient–dentist relationship; Agreed treatment plan	Provider bias in offering treatment; Dentist competence/experience; Clinician decision-making responsibility	Comprehensive evidence-based treatment planning; Dentist competence/experience; Decision aids/shared decision making tools; Patient factors shaping provider's decision; Shared decision-making
Al-Mashhadani S, Nasser M, Alsalami A, Burns L, & Paisi M. (2024) [20]	Barriers and Facilitators to Dental Care Services Utilization Among Children With Disabilities: A Systematic Review and Thematic Synthesis	United Kingdom & United Arab Emirates	Children with Disabilities	Limited access to reliable information; Perceived provider competence; Communication barriers; Parental past negative experiences; Stigma; Gender stereotyping	Respect and autonomy; Effective communication and trust; Accommodations for communication; Parent/caregiver education; Availability of resources; Patient- and family-centred individualized, holistic approach; Cultural considerations	Lack of knowledge, training, or skills; Provider stigma/reluctance; Communication barriers; Lack of transparent information or referral systems/multidisciplinary access; Inadequate compensation for efforts	Robust communication systems; Training and professional development; Mentorship and access to specialists/multidisciplinary collaboration; Availability of resources
Alharkan HM. (2024) [21]	Integrating digital smile design into restorative Dentistry: A narrative review of the applications and benefits	Saudi Arabia	Restorative Dentistry; Digital Smile Dentistry	Emotional impact/insecurities and heightened anxiety; Unrealistic/unmet expectations	Transparent communication; Involvement and empowerment in decision-making; Esthetic and functional outcomes; Technological tools	Learning curve/new technical skills; Technological tools	Digital Smile Design; Treatment planning
Asa’ad F. (2019) [22]	Shared decision‐making (SDM) in dentistry: A concise narrative review	Sweden	Shared-Decision Making; General Dentistry	Anxiety; Low oral health literacy, misunderstandings, beliefs	Shared decision-making; Tailored/individualized information; Patient decision aids	Difficulty implementing shared decision-making; Difficulty obtaining informed consent; Mismatched provider–patient treatment preferences	Training and professional development; Shared decision making; Decision aids/shared decision making tools
Campbell SD, Cooper L, Craddock H, Hyde TP, Nattress B, Pavitt SH, & Seymour DW. (2017) [23]	Removable partial dentures: The clinical need for innovation	United States of America & United Kingdom	Patients with Partial Edentulism; Removable Partial Dentures	Pain; Dissatisfaction with removable partial dentures; Esthetic preferences	Preference-aligned design (esthetics & comfort); Patient education	Provider focus misaligned with patient concerns	Digital dentistry
Cardoso M, Coelho A, Lima R, Amaro I, Paula A, Marto CM, Sousa J, Spagnuolo G, Marques Ferreira M, & Carrilho E. (2020) [24]	Efficacy and Patient’s Acceptance of Alternative Methods for Caries Removal – A Systematic Review	Portugal, Italy, & Russia	Alternative Methods for Caries Removal	Anxiety; Fear; Pain; Aversive sensory experiences	Alternative methods	Techniques lacking familiar tactile or sensory feedback; Laser therapy or air-/sono-abrasion	Provider confidence in familiar techniques
Dahlan R, Badri P, Saltaji H, & Amin M. (2019) [25]	Impact of acculturation on oral health among immigrants and ethnic minorities: A systematic review	Canada	Immigrants and Ethnic Minorities		Presence of an interpreter		
Dalsochio L, Montagner AF, Tedesco TK, Maske TT, & van de Sande FH. (2025) [26]	Experiences and Parents' Perceptions Regarding Dental Interventions Performed on Their Children: A Qualitative Systematic Review	Brazil	Parents' Perceptions; Pediatric Dental Interventions	Anxiety; Fear; Past negative experiences; Beliefs; Low perceived need or priority; Low oral health literacy; Reluctance toward invasive treatment; Esthetic/material preference; Perceived provider competence; Communication and interpersonal care; Embarrassment/guilt; Reliance on home remedies or self-medication; Concerns about adequacy of minimally invasive strategies; Busy family life	Trust; Communication and interpersonal care; Perceived provider competence; Oral health guidance and educational materials; Preference for minimally invasive dentistry; Familiar/school settings; Engagement strategies and child friendly tools; Counselling		
da Silva Ribeiro Júnior H, de Brito BA, & Corrêa-Faria P. (2024) [27]	Parents' acceptance of minimal intervention procedures for dental caries management in children: a scoping review	Brazil	Parents' Perceptions (for their children); Minimal Intervention Procedures for Dental Caries Management	Esthetic/material preference; Duration of procedure	Esthetic preference/less visible placement; Comfort, painlessness, and acceptable taste; Avoidance of sedation, general anaesthesia and injections		
Datta S, Sybil D, & Jain V. (2020) [28]	Teledentistry Applications: Potential Dental Care Facilitator amidst a Pandemic	India	Teledentistry Applications	Mistrust/uncertainty about teledentistry advice	Patient acceptance/positive reception of care modality; Educational/instructional materials; Technological tools	Technological tools	Teledentistry
Dyer TA, & Robinson PG. (2016) [29]	The acceptability of care provided by dental auxiliaries: A systematic review	United Kingdom	Dental Auxiliaries	Reluctance toward dental auxiliary care (especially for children); Perceived provider competence; Beliefs/values	Acceptability of/satisfaction with dental auxiliary care; Trust (in the dentist/team and in training/regulation); Perceived provider competence; Communication and interpersonal care; Continuity of care		
Edelmayer M, Woletz K, Ulm C, Zechner W, & Tepper G. (2016) [30]	Patient information on treatment alternatives for missing single teeth - Systematic Review	Austria	Missing Single Teeth	Fear; Procedural risk/safety concerns; Low oral health literacy/perceived knowledge deficit; Long duration of procedure	Patient information and education; Preference for fixed implant options (over removable dentures)		
Fägerstad A, Windahl J, & Arnrup K. (2016) [31]	Understanding avoidance and non-attendance among adolescents in dental care - an integrative review	Sweden	Adolescents	Anxiety; Fear; Pain/painful experiences; Negative attitudes toward the dentist; Low perceived need or priority; Discontinuities in care			
Fakhrjahani I, Tiwari T, & Jessani A. (2024) [32]	A Scoping Review of Oral Health Outcomes and Oral Health Service Utilization of 2SLGBTQ+ People	Canada & United States of America	2SLGBTQ+ Individuals	Stigma and discrimination; Misgendering; Perceived provider competence; Fear; Psychosocial factors	Empathetic care; Urgency-driven care seeking		
Frey-Furtado L, Fonseca M, Melo P, Listl S, & Pereira ML. (2025) [33]	Oral healthcare access: self-perceived barriers faced during pregnancy - a systematic review	Portugal, Netherlands, & Germany	Pregnant Women	Fear; Anxiety; Beliefs; Low oral health literacy/limited awareness; Perceived provider competence/mistrust; Cultural beliefs and misguided family/friend advice; Physical discomfort during care; Lack of recommendations and interdisciplinary collaboration	Patient support for oral health education and integration into prenatal care	Limited interdisciplinary collaboration	
Gambhir RS, Brar P, Singh G, Sofat A, & Kakar H. (2013) [34]	Utilization of dental care: An Indian outlook	India		Anxiety; Fear; Beliefs/myths; Low perceived need or priority; Reliance on home remedies, self-medication, or traditional medicine; Past negative experiences; Lack of time	Education		
Hu S, Meyer B, & Duggal M. (2018) [35]	A silver renaissance in dentistry	Singapore & United States of America	Silver Diamine Fluoride	Esthetic concerns (silver diamine fluoride black staining)	Preference-aligned procedures		Low-demand, minimally invasive alternative (SDF)
Ismail AF, Tengku Azmi TMA, Malek WMSWA, & Mallineni SK. (2021) [36]	The effect of multisensory-adapted dental environment on children's behavior toward dental treatment: A systematic review	Malaysia & Saudi Arabia	Children		Sensory-adapted dental environment		Sensory-adapted dental environment
Ismail N, Mohd Yusof MYP, & Wan Mokhtar I. (2024) [37]	Factors influencing parental acceptance toward the use of passive immobilisation as behaviour guidance in children during dental treatment: a scoping review	Malaysia	Parents' Perceptions (for their children); Children with Special Healthcare Needs	Aversive perception of passive immobilisation; Anxiety; Cultural beliefs/background	Information & exposure method (explanation/demonstration increasing acceptance); Prior experience & trust; Urgency-driven acceptance		Informed consent
Järvinen M, Stolt M, Honkala E, Leino-Kilpi H, & Pöllänen M. (2018) [38]	Behavioural interventions that have the potential to improve self-care in adults with periodontitis: a systematic review	Finland & Norway	Adults; Periodontitis		Patient valued educational/behavioural support		
Jawdekar A, Tafti F, Deolikar S, & Mistry L. (2024) [39]	Acceptance of Parents toward Hand-over-mouth Exercise and Other Behavior Management Techniques for Pediatric Dental Care in the 21st Century: A Systematic Review and Meta-analysis of Observational Studies	India	Parents' Perceptions (for their children)	Aversive behaviour management techniques (low acceptance of hand-over-mouth exercise); Fear of treatment risk (general anaesthesia)	Preference for communicative/non aversive techniques		
Jugé C, & Tubert-Jeannin S. (2013) [40]	Effets de l'hypnose lors des soins dentaires [Effects of hypnosis in dental care]	France	Hypnosis		Hypnosis		
Kamalabadi YM, Campbell MK, Zitoun NM, & Jessani A. (2023) [41]	Unfavourable beliefs about oral health and safety of dental care during pregnancy: a systematic review	Canada	Pregnant Women (or mothers with children under 6 years of age)	Low oral health literacy; Beliefs/myths; Cultural beliefs and misguided family/friend advice; Low perceived need or priority; Dental fatalism	Education		
Kashbour WA, Rousseau NS, Ellis JS, & Thomason JM. (2015) [42]	Patients' experiences of dental implant treatment: A literature review of key qualitative studies	United Kingdom	Dental Implant Treatments	Anxiety; Fear of pain and surgical distress; Past negative experiences; Perceived treatment risks and burden; Discomfort/drawbacks of implant restorations	Desire for normalisation and restored identity; Functional and social improvement; Trust		
Kupzyk S, & Allen KD. (2019) [43]	A Review of Strategies to Increase Comfort and Compliance with Medical/Dental Routines in Persons with Intellectual and Developmental Disabilities	United States of America	Persons with Intellectual and Developmental Disabilities		Behavioural supports		
Lapidos A, Shaefer HL, & Gwozdek A. (2016) [44]	Toward a better understanding of dental appointment-keeping behavior	United States of America		Anxiety; Fear; Dental staff attitudes; Low perceived need or priority; Family or caregiving responsibilities; Forgetfulness; Transportation difficulties and time constraints	Case manager support		
Lechte C, Schlarmann F, Biermann J, Wiegand A, & Kanzow P. (2024) [45]	Repair of partially defective restorations: Systematic review and meta-analysis of patient acceptance	Germany	Partially Defective Restorations		Procedural preference		Provider belief in high patient acceptance (enabling repair provision)
Lin GSS, Cher CY, Cheah KK, Koh SH, Chia CHL, Lim VR, Baharin F, & Wafa S WWSST. (2022) [46]	Acceptability of atraumatic restorative treatment and Hall Technique among children, parents, and general dental practitioners: a systematic review and meta-analysis	Malaysia	Children & Parents; Atraumatic Restorative Treatment and Hall Technique	Esthetic concerns (Hall Technique metal crown appearance)	Preference for minimally invasive/non aversive procedures; Preference aligned by treatment comparison (atraumatic restorative treatment vs Hall Technique vs conventional)	Reluctance toward atraumatic restorative treatment	Provider preference for less technically demanding technique (Hall Technique > atraumatic restorative treatment)
Magno MB, Silva LPD, Ferreira DM, Barja-Fidalgo F, & Fonseca-Gonçalves A. (2019) [47]	Aesthetic perception, acceptability and satisfaction in the treatment of caries lesions with silver diamine fluoride: A scoping review	Brazil	Treatment of Caries Lesions with Silver Diamine Fluoride	Esthetic concerns (silver diamine fluoride staining, anterior teeth)	Trust; Willingness to accept esthetic trade-offs and procedural preference	Provider preconception of parental non-acceptance	
Marshall A, Loescher A, & Marshman Z. (2016) [48]	A scoping review of the implications of adult obesity in the delivery and acceptance of dental care	United Kingdom	Obese or Overweight Adults	Anxiety; Embarrassment; Stigma/self-consciousness		Specialized equipment and personnel	Specialist collaboration
Mazurat NM, & Mazurat RD. (2003) [49]	Discuss Before Fabricating: Communicating the Realities of Partial Denture Therapy. Part 1: Patient Expectations	Canada	Partial Denture Therapy	Low perceived need/priority; Esthetic preferences/concerns	Functional and esthetic benefits		
Monteiro J, Tanday A, Ashley PF, Parekh S, & Alamri H. (2020) [50]	Interventions for increasing acceptance of local anaesthetic in children and adolescents having dental treatment	United Kingdom & Saudi Arabia	Children & Adolescents; Local Anesthetic in Dental Treatment	Fear/anxiety of dental injections	Behavioural interventions		
Outhaisavanh S, Liu Y, & Song J. (2020) [51]	The origin and evolution of the Hawley retainer for the effectiveness to maintain tooth position after fixed orthodontic treatment compare to vacuum-formed retainer: A systematic review of RCTs	China	Fixed Orthodontic Treatment & Retainers	Esthetic concerns/embarrassment (Hawley retainer metallic appearance)	Preference-aligned procedures		
Patil MS, & Patil SB. (2009) [52]	Geriatric patient - psychological and emotional considerations during dental treatment	India	Geriatric Individuals	Anxiety; Embarrassment, stigma & self-image concerns (tooth loss)	Education		
Pisano M, Bramanti A, Di Spirito F, Di Palo MP, De Benedetto G, Amato A, & Amato M. (2025) [53]	Reviewing Mobile Dental Apps for Children with Cognitive and Physical Impairments and Ideating an App Tailored to Special Healthcare Needs	Italy	Children with Cognitive and Physical Impairments	Anxiety & fear (dental setting/unfamiliarity); Aversive sensory experiences; Stigma/discrimination; Caregiver fear of causing harm & low confidence; Digital navigation difficulties; Lack of support	Child engagement & enjoyment (digital tools); Caregiver acceptance & reduced stress (digital tools)	Insufficient training/skills (provider preparedness for special health care needs)	Provider acceptance of tailored digital tools
Pritchard L, Burden KJ, Carlson-Jones W., Stormon N, & Do L. (2025) [54]	Implementation and Acceptance of Oral Health Assessment Tools in Residential Aged Care Facilities: A Scoping Review	Australia	Individuals in Residential Aged Care	Embarrassment/appearance concerns; Discomfort/fear-driven care resistance	Autonomy/choice & clear explanation	Negative attitudes, low prioritisation & reluctance; Insufficient training/skills & low confidence	Provider acceptance of assessment tools
Ribeiro CDPV, Alves JB, Kominami PA, Takeshita EM, Bezerra ACB, & Massignan C. (2023) [55]	Does use of animal therapy during dental care help to reduce anxiety in children and adolescents? A systematic review	Brazil	Children & Adolescents		Animal-assisted therapy (inconclusive)		
Rocha JS, Arima LY, Werneck RI, Moysés SJ, & Baldani MH. (2018) [56]	Determinants of Dental Care Attendance during Pregnancy: A Systematic Review	Brazil	Pregnant Women	Low perceived need or priority; Beliefs; Psychological factors			
Sondell K, & Söderfeldt B. (1997) [57]	Dentist-patient communication: a review of relevant models	Sweden			Patient desire for information-sharing/non-dominant communication		
ter Horst G, & de Wit CA. (1993) [58]	Review of behavioural research in dentistry 1987-1992: dental anxiety, dentist-patient relationship, compliance and dental attendance	Netherlands	Behavioural Research in Dentistry	Anxiety; Fear; Perceived provider competence/attitude & communication; Embarrassment	Preference for calm, reassuring, informative provider behaviour; Preference for introductions and clear explanation of procedures/costs; Behavioural interventions	Insufficient training/skills & equipment	Provider willingness
Touati R, Sailer I, Marchand L, Ducret M, & Strasding M. (2022) [59]	Communication tools and patient satisfaction: A scoping review	France & Switzerland			Preference-aligned by consultation-method comparison (visual/digital tools)		
Valdez R, Spinler K, Kofahl C, Seedorf U, Heydecke G, Reissmann DR, Lieske B, Dingoyan D, & Aarabi G. (2022) [60]	Oral Health Literacy in Migrant and Ethnic Minority Populations: A Systematic Review	Germany	Migrant & Ethnic Minorities	Beliefs; Low perceived need/problem-oriented attendance; Reliance on home remedies/traditional medicine	Beliefs; Culturally sensitive approach		
Valentim FB, Moreira KMS, Carneiro VC, do Nascimento LJ, Colares V, & Imparato JCP. (2023) [61]	Cost-effectiveness and Acceptance in Children and Parents of the Hall Technique: Systematic Review of Clinical Trials	Brazil	Parents' Perceptions (for their children)	Anxiety (aversive steps: anaesthesia/needle, drill noise, long procedure); Pain/discomfort; Esthetic concerns (Hall Technique metal crown appearance)	Preference-aligned by treatment comparison (Hall Technique preferred; non-aversive/quick); Willingness to accept esthetic tradeoff/child esthetic acceptance of Hall Technique; Trusting and continuous relationships		Cost effective procedures with shorter chairtime (Hall Technique)
Vundavalli S, Indiran MA, Doppalapudi R, Siddanna S, Baig MN, Issrani R, & Prabhu N. (2025) [62]	Barriers to Dental Services Utilization among Adult Population in India: A Scoping Review	India & Saudi Arabia	Adults	Fear; Low perceived need or priority; Cultural beliefs/myths; Low oral health literacy/lack of awareness; Reliance on home remedies/self-medication			
Wilson A, Hoang H, & Barnett T. (2021) [63]	Barriers and enablers to skill-mix in the oral health workforce: A systematic review	Australia	Skill Mix	Reluctance toward dental auxiliary care (especially for children); Anxiety; Beliefs/values	Acceptability of/satisfaction with dental auxiliary care; Trust; Perceived provider competence; Communication and interpersonal care; Continuity of care; Education; Cost-effective dental care	Provider reluctance/negative attitudes toward dental care professionals; Dentist doubts about dental care professional clinical competence/quality; Lack of clinical space; Remuneration and reimbursement issues	Provider support/acceptance of skill-mix (dentists)
Zhou Y, Cameron E, Forbes G, & Humphris G. (2011) [64]	Systematic review of the effect of dental staff behaviour on child dental patient anxiety and behaviour	United Kingdom	Dental Staff Behaviour; Children		Empathetic working style	Provider non-acceptance of assertive technique (voice control)	
Freeman R, Lush C, MacGillveray S, Themessl-Huber M, & Richards D. (2013) [65]	Dental therapists/hygienists working in remote-rural primary care: a structured review of effectiveness, efficiency, sustainability, acceptability and affordability	United Kingdom				Provider reluctance/negative attitudes toward dental therapists/hygienists	Provider support/acceptance of dental therapists/hygienists (dentists)
Grey Literature							
Canadian Dental Association (CDA) (2017) [66]	CDA Essentials	Canada	Children with Special Needs, Vulnerable people; Silver Diamine Fluoride	Perceived provider competence	Perceived provider competence; Public dental coverage; Procedural preference	Provider reluctance/inability to treat special health care needs; Low reimbursement	Compensation when extra time and effort is required
Flynn B, Weninger Starkel R, Zaborowski M, Vujicic M; American Dental Association (ADA) (2024) [67]	Barriers to Dental Care Among Adult Medicaid Beneficiaries: A Comprehensive Analysis in Eight States	United States of America	Adult Medicaid Beneficiaries	Fear; Anxiety; Low perceived need or priority; Low oral health literacy; Cultural and linguistic misalignment; Stigma; Time constraints	Mental health support	Negative provider perceptions/attitudes toward Medicaid beneficiaries; Provider stigma (Medicaid participation/peer reputation); Low reimbursement and administrative burden	
National Health Service (NHS) England (2018) [68]	A needs assessment for General Dental Services in Kent, Surrey and Sussex	United Kingdom		Anxiety; Low perceived need; Mistrust; Availability of suitable services; Long travel times	Preference for accepting, non-judgmental care setting		
National Health Service (NHS) UK (2019) [69]	Skills for Health Dental Nurse Apprenticeship Standard Survey Health Education England Organisational Response	United Kingdom		Beliefs/values; Reluctance toward dental auxiliary care/low awareness of dental care professional role	Acceptability of/satisfaction with dental auxiliary care; Perceived provider competence/communication & time taken; Preference-aligned by provider comparison (preference for hygienist/therapist over dentist)	Provider poor attitude/reluctance toward dental care professionals	Provider valuing of dental care professionals as team members (willingness to delegate)
Pan American Health Organization (PAHO) (2010) [70]	Workshop on Caries Prevention for Communities in Belize	International	Caries Prevention		Cultural, family-based care		
Public Health England (2021) [71]	Inequalities in oral health in England	United Kingdom		Anxiety; Fear; Past negative experiences; Low oral health literacy; Cultural and linguistic factors; Low perceived need or priority; Dental fatalism; Mistrust; Dental staff attitudes; Managing work and family responsibilities; Long wait times and unsuitable appointment timing; Shortage of dentists	Flexible scheduling	Insufficient training, skills & confidence + reluctance to treat; Limited collaboration	Financial incentives within healthcare remuneration systems
Public Health England (2025) [72]	Oral care and people with learning disabilities	United Kingdom	People with Learning Disabilities	Anxiety; Aversive sensory experiences; Past negative experiences; Communication challenges; Dental staff attitudes; Transition between children and adult services; Forgetfulness; Limited mobility	Information/preparation for visit; Trusting and continuous relationships; Shared decision-making; Behavioural interventions; Sensory aids	Insufficient training, confidence and knowledge; Inadequate reimbursement structures	Sensory aids; Training
Australian Institute for Health and Welfare (AIHW) (2020) [73]	Cultural competency	Australia	Indigenous Australians	Fear; Embarrassment; Cultural and linguistic misalignment; Dislike of service or provider; Mistrust; Perceived inadequacy of care; Discrimination	Continuity of care; Culturally competent care; Provider competence and communication		
Australian Institute for Health and Welfare (AIHW) (2025) [74]	Oral health and dental care in Australia	Australia		Lack of dental health professionals with skills in special-needs dentistry; Long treatment times and limited transportation options			
Junger ML, Griffin SO, Lesaja S, Espinoza L.; Centers for Disease Control and Prevention (CDC) (2019) [75]	Awareness Among US Adults of Dental Sealants for Caries Prevention	United States of America	Adults; Dental Sealants for Caries Prevention	Low oral health literacy			
Yeung CA, Donachie M, Dow S, Hall A.; National Health Service (NHS) Scotland (2012) [76]	Restorative Dentistry: Needs Assessment Report	United Kingdom	Restorative Dentistry		Sense of safety/comfort; Acceptance of student/trainee care (with supervision & explanation); Patient-centred care/involvement/feeling respected; Value placed on natural teeth/reluctance to accept tooth loss	Capacity limitations	Provider support/acceptance of skill-mix (managed clinical network delegation models); General dental practitioner value/trust in specialist service; Electronic referral systems
Canadian Academy of Health Sciences (CAHS) (2014) [77]	Improving Access To Oral Health Care For Vulnerable People Living In Canada	Canada	Vulnerable People	Aversion to amalgam/mercury + preference for tooth-coloured materials; Jurisdictional variations	Preference for public/community care settings over private	Negative provider perceptions/reluctance to treat publicly-insured/low-income patients	
FDI World Dental Federation (2005) [78]	Point of Care	Canada		Anxiety; Fear; Past negative experiences		Provider dislike/reluctance toward treating dentally anxious patients	
Government UK (2024) [79]	Adult oral health survey 2021: service use and barriers to accessing care	United Kingdom	Adults	Anxiety; Low perceived need or priority; Previous negative experiences; Forgetfulness; Time constraints			
NHS England South East (2019) [80]	Personal Dental Services Specialist-led Special Care and Paediatric Dentistry Draft Service Specification	United Kingdom	Paediatric Dentistry				
Gondro JV, Murphy K, Clark J, Fortin Y; Statistics Canada (2024) [81]	Factors associated with the use of oral health care services among seniors in Canada	Canada	Seniors	Fear; Low perceived need or priority			
Murphy K, Gondro JV, Moharrami M; Statistics Canada (2024) [82]	Factors associated with the use of oral health care services among Canadian children and youth	Canada	Children and Youth				

An abductive thematic analysis [83] was applied within a constructivist/interpretivist paradigm. This approach was chosen as acceptability in oral health care is not yet well-defined, requiring both theoretical grounding and flexibility to capture themes that existing frameworks may not account for. Existing healthcare access and acceptability frameworks [8, 9] informed the initial interpretive lens, while the research team’s disciplinary backgrounds and experiential knowledge in dental practice allowed for the recognition of new themes specific to the oral health care context. Extracted findings were coded and categorized through iterative discussions, moving between framework-informed categories and patterns emerging from the data until final themes were established.

### Quality assessment

Consistent with scoping review methodology, no formal quality appraisal was conducted, as reviews of varying rigor may still contribute relevant insights [18].

## Results

### Characteristics of included literature

The database search yielded 221 records, with 204 remaining after de-duplication. Following title and abstract screening, 121 articles underwent full-text review, of which 74 were excluded (55 had an ineligible study design, 17 had a topic that was not relevant, 1 was a duplicate or a superseded review, and 1 was withdrawn). A total of 47 peer-reviewed reviews were included [19–65]. These reviews, published between 1993 and 2025, were authored by researchers from Australia [54, 63], Austria [30], Brazil [26, 27, 47, 55, 56, 61], Canada [25, 32, 41, 49], China [51], Finland [38], France [40, 59], Germany [33, 45, 60], India [28, 34, 39, 52, 62], Italy [24, 53], Malaysia [36, 37, 46], Netherlands [33, 58], Norway [38], Portugal [24, 33], Russia [24], Saudi Arabia [19, 21, 36, 50, 62], Singapore [35], Sweden [22, 31, 57], Switzerland [59], United Arab Emirates [20], United Kingdom (UK) [19, 20, 23, 29, 42, 48, 50, 64, 65], and the United States of America [23, 32, 35, 43, 44]. Specific populations studied included adolescents [31, 50, 55], adults (general [38, 62] and those who are overweight or obese [48]), children (general [26, 27, 36, 37, 39, 46, 50, 55, 61] and those with disabilities [20, 53]), persons with intellectual and developmental disabilities [43], migrants and ethnic minorities [25, 60], older adults [52], pregnant individuals [33, 41, 56], and 2SLGBTQ+ people [32]. Specific oral health treatments included caries management [24, 47], implants [19, 42], minimally invasive dentistry [26], local anesthetics [50], treatment for missing single teeth [30], orthodontics [51], partial dentures [23, 49], periodontitis [38], restorative dentistry [21, 45, 46], silver diamine fluoride (SDF) [35, 47], and teledentistry [28].

The grey literature search yielded 735 webpages; 17 eligible sources published between 2005 and 2025 were included [66–82]. They were affiliated with the following organizations: American Dental Association [67], Australian Institute for Health and Welfare [73, 74], Canadian Academy of Health Sciences [77], Canadian Dental Association [66], Centers for Disease Control and Prevention [75], World Dental Federation [78], Government of UK [79], National Health Service England [68, 80], National Health Service Scotland [76], National Health Service UK [69], Pan American Health Organization [70], Public Health England [71, 72], and Statistics Canada [81, 82]. The organizations were from the following countries: Australia [73, 74], Canada [66, 77, 81, 82], United Kingdom [68, 69, 71, 72, 76, 79, 80], the United States of America [67, 75], and international [70]. Populations included adults (general [75, 79] and Medicaid beneficiaries [67]), pediatrics (general [80, 82] and with special needs [66]), Indigenous populations [73], people with learning disabilities [72], seniors [81], and vulnerable people [66, 77]. Specific oral health treatments included caries management [70, 75], restorative dentistry [76], special care dentistry [80], and SDF [66].

**Fig. 1:**
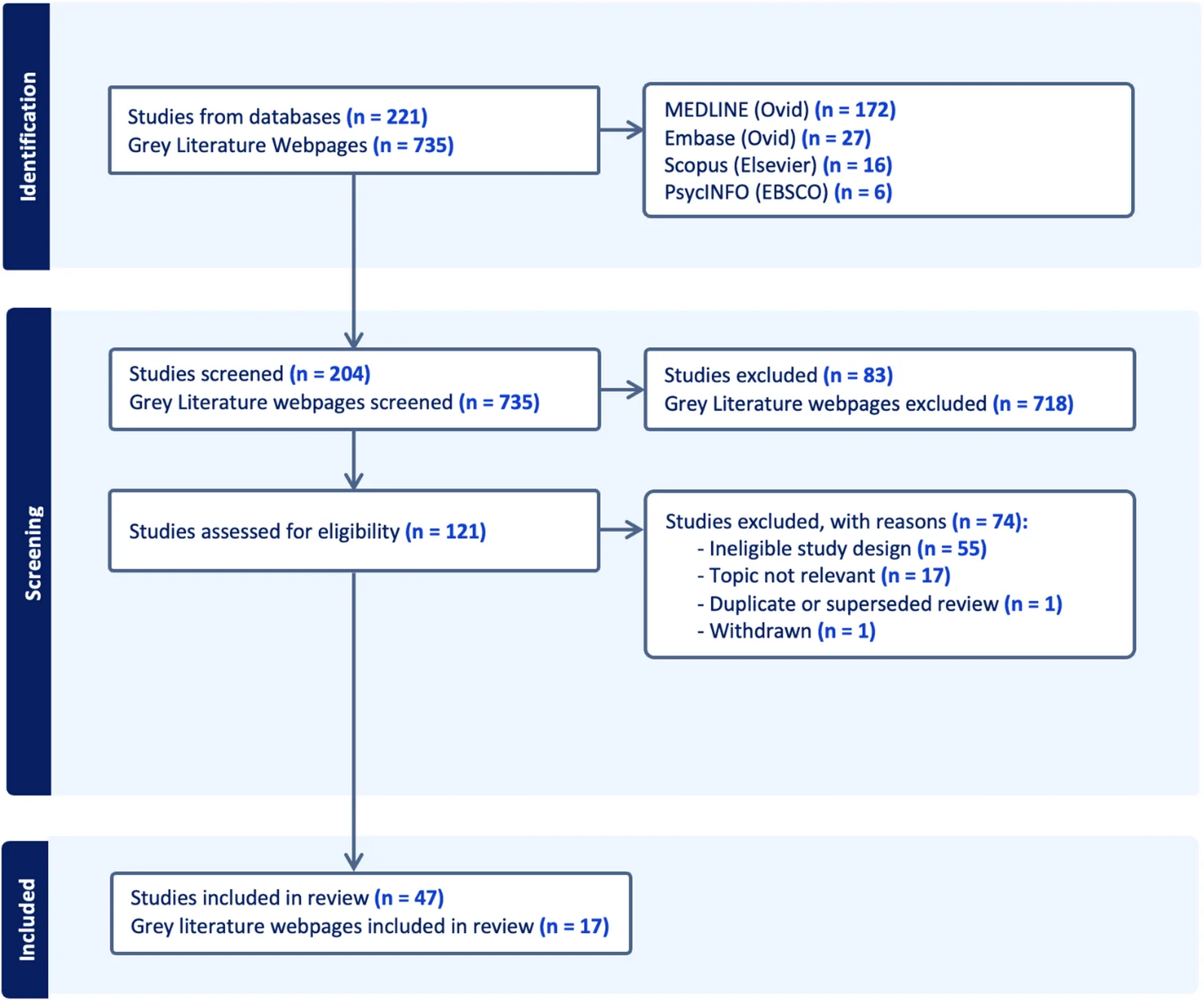
PRISMA flow diagram of the study selection process

The breakdown of the search process is represented in Fig. 1.

## Findings

**Fig. 2:**
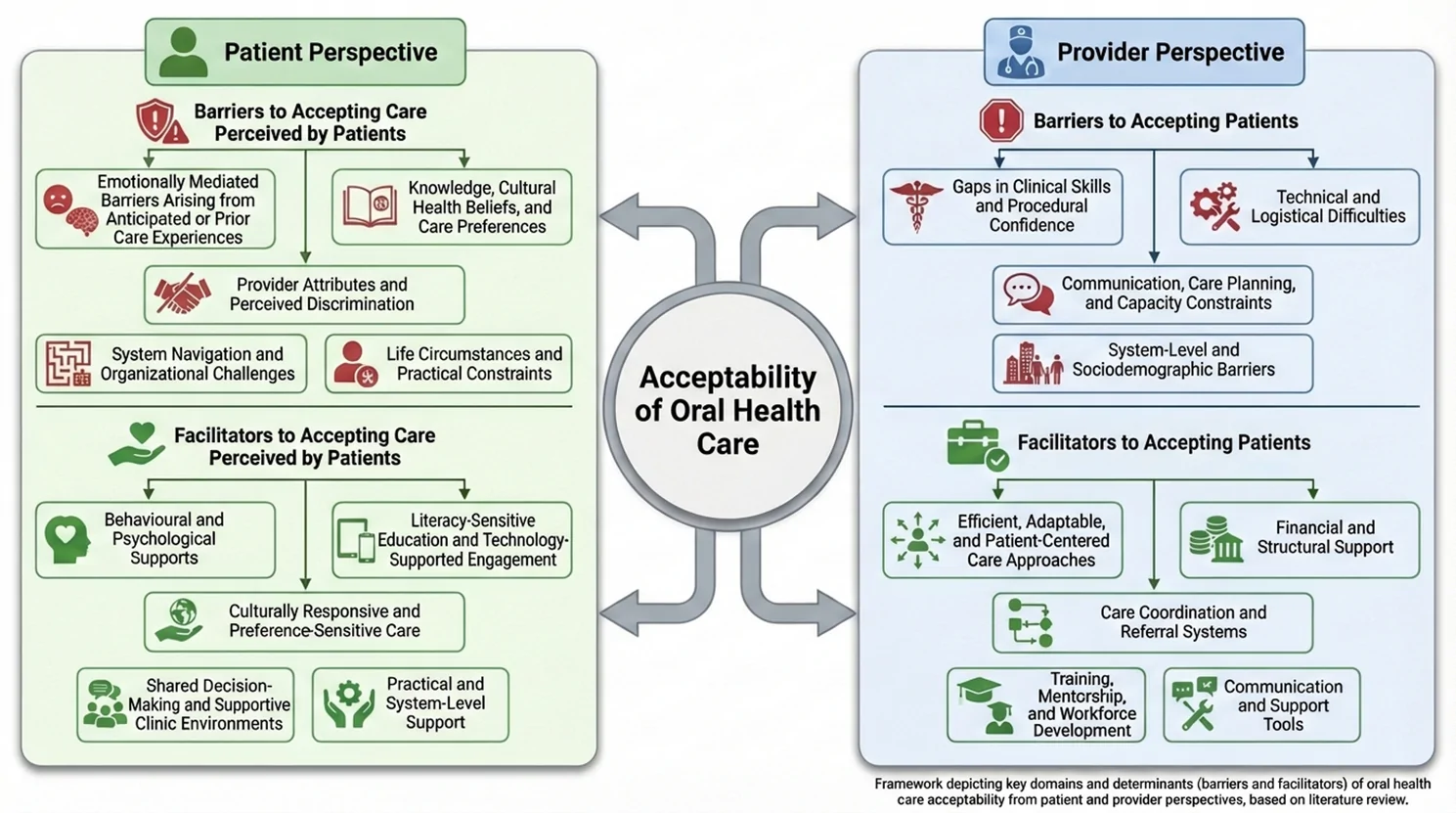
Acceptability of oral health care framework

The findings below are organized into two major dimensions: the patient perspective and the provider perspective. Within each perspective, barriers and facilitators were identified, and each section was further structured into themes. The framework for oral health care acceptability, developed by mapping the various themes identified under the two dimensions, is presented in Fig. 2.

### Patient perspectives

#### Barriers to accepting care perceived by patients

##### Emotionally mediated barriers arising from anticipated or prior care experiences

Anxiety was the most frequently reported determinant of non-uptake or disengagement [19, 21, 22, 24, 26, 31, 33, 34, 37, 42, 44, 48, 50, 52, 53, 58, 61, 63, 67, 68, 71, 72, 78, 79], followed by fear, including dental phobia and fear of specific stimuli such as rotary instruments [19, 24, 26, 30, 31, 32, 33, 34, 39, 42, 44, 50, 53, 54, 58, 62, 67, 71, 73, 78, 81]. Discomfort during dental procedures also negatively affected acceptability [33, 42, 54, 61]. Concerns about pain or painful procedures were common [19, 23, 24, 31, 42, 61]. Sensory aspects of dental care including vibration, noise, taste, smell, and altered oral sensations, contributed to avoidance and heightened distress [24, 53, 72]. Embarrassment was another emotional factor reported among individuals with poor dental appearance, including older adults, adults with obesity, and Indigenous peoples [26, 48, 51, 52, 54, 58, 73]. Some reviews found procedure related concerns: for example, for invasive or complex procedures, patients shared safety or complication related concerns or fear of harm or side-effects, affecting acceptance [19, 26]. Similarly, dissatisfaction with removable partial dentures contributed to underuse and rehabilitation failure [23]. 

##### Knowledge, cultural health beliefs, and care preferences

Patients’ understanding of oral health, shaped by knowledge, cultural beliefs, and personal values, played a central role in determining the acceptability of oral health care. Low oral health literacy and a general lack of knowledge or awareness were pervasive across multiple populations and influenced how individuals interpreted oral health needs and engaged with services [22, 26, 30, 33, 41, 62, 67, 69, 71, 75]. Patient perceived cultural and linguistic misalignment with oral health services, including providers’ lack of cultural safety or sensitivity and language barriers, limited engagement and acceptability of care across several studies [33, 37, 41, 62, 67, 71, 73]. Beliefs, including myths and misconceptions about dental diseases and treatment safety, shaped negative attitudes and preferences toward oral care [22, 26, 29, 33, 34, 41, 56, 60, 62, 63, 69]. Lower perceived need or priority, and self-assessments of oral health were consistently associated with non-utilization of services [26, 31, 34, 41, 44, 49, 56, 60, 62, 67, 68, 71, 79, 81]. In several populations, reliance on home remedies or over-the-counter medications reflected prevailing beliefs and preferences and often delayed professional care-seeking [26, 34, 60, 62]. Dental fatalism, a deterministic belief among children’s caregivers that dental decay is largely inevitable regardless of preventive or care efforts, was identified as a barrier to regular dental check-ups, as this belief diminishes motivation for maintaining children’s routine oral health care, including regular visits to a dental professional [41, 71]. Preferences related to care delivery also influenced acceptability. Certain behaviour management techniques for children, such as passive immobilization and the hand-over-mouth exercise were viewed as less acceptable [37, 39]. Esthetic priorities also influenced preferences for specific dental procedures or materials [23, 26, 27, 35, 46, 47, 49, 51, 61], while more invasive procedures were often less preferred [19, 26].

##### Provider attributes and perceived discrimination

Perceived gaps in provider training or skills, lack of support, and misalignment with patient priorities reduced acceptability of care, particularly when patients felt that clinicians lacked the competencies needed to meet their specific needs [20, 26, 29, 32, 33, 53, 58, 63, 66, 69]. Concerns were especially prominent for child populations, including reluctance toward dental auxiliary care for pediatric patients [63] and a lack of specialized dentistry [74]. Dislike of the service or provider, along with feelings of embarrassment or fear and perceptions that care would be inadequate, further reduced acceptability among Indigenous patients [73]. Mistrust, often stemming from prior negative experiences, was a recurrent theme shaping ongoing perceptions of acceptability [28, 33, 68, 71, 73]. Patients described experiencing negative or dismissive attitudes from dental staff, which created emotional discomfort and discouraged care-seeking [44, 58, 71, 72]. Communication challenges, including miscommunication, misunderstandings, or difficulty expressing needs were repeatedly linked to lower acceptability [20, 22, 26, 58, 72]. For some groups, frustration arose from feeling excluded from treatment planning, particularly among patients with disabilities and their caregivers who perceived insufficient communication from providers [20]. Paternalistic provider-patient dynamics further contributed to feelings of disempowerment [19].

Discrimination and stereotyping also played a significant role in lowering acceptability. Patients reported experiences of gender stereotyping, where assumptions about gender influenced the care provided or how seriously concerns were taken, which could lead to feelings of being undervalued or dismissed [20]. Misgendering, or being referred to with incorrect gender pronouns, was reported by transgender or gender-diverse patients and contributed to emotional discomfort, distrust, and reluctance to engage with care [32]. More broadly, patients experienced stigma or discriminatory treatment related to age, ethnicity, socioeconomic status, or other personal characteristics, which reinforced mistrust, reduced satisfaction, and discouraged future care-seeking [20, 32, 48, 52, 53, 67, 73].

##### System navigation and organizational challenges

System-level discontinuities, including challenges during care transitions, complex referral pathways, poor continuity of care, weak integration with other health and social services, and jurisdictional ambiguities were repeatedly identified as barriers to the acceptability of oral health care [31, 33, 72, 77]. From the patient perspective, these organizational features generated confusion, delays, and frustration, reducing engagement with care even when services were available and affordable [31, 33, 72, 77]. Digital-navigation difficulties were also reported: for example, users experienced technical problems with dental mobile-health applications, such as connectivity issues or failures in recording brushing data, which interfered with care processes and diminished acceptability of digitally supported oral health interventions [53]. 

##### Life circumstances and practical constraints

Patients’ broader life circumstances play a significant role in shaping the acceptability of oral health care. Personal and family challenges including family structure, limited support, parenting demands, and wider social influences affected motivation and ability to engage with care [26, 44, 53, 71]. Exhaustion or burnout from family or caregiving responsibilities was noted to affect appointment-keeping behaviours [44]. Psychosocial pressure and prevailing social norms further impeded uptake [32, 56]. Alongside these social and personal influences, time and logistical demands presented substantial barriers. Long waiting times, lengthy procedures, transportation difficulties, restricted clinic hours, and competing daily obligations limited patients’ capacity to attend or complete care [27, 30, 34, 44, 67, 68, 71, 74, 79]. Limited mobility [72] and physiological limitations [33], and inadequate proximity or availability of services [68] were frequently reported. Provider shortages and difficulty finding a dentist compounded logistical barriers [71, 74]. Missed appointments were attributed to difficulty in scheduling [71] and forgetfulness [44, 72, 79]. These personal and logistical attributes overlap across different dimensions of access to care, including availability of providers, physical accessibility to care, and accommodation in scheduling needs; ultimately, all influencing acceptability of care.

#### Facilitators to accepting care perceived by patients

##### Behavioural and psychological supports

A range of behavioural and psychological strategies support patients in managing anxiety, fear, and distress during dental care. Non-pharmacological and behavioural techniques, including positive reinforcement, distraction, modelling, hypnosis, cognitive behavioural therapy, and conscious sedation could reduce anxiety and improve cooperation across various ages [38, 40, 43, 50, 58, 72]. Access to mental health supports further addressed dental anxiety and improved care experiences [67]. Sensory-adapted environments, sensory aids, and other reasonable adjustments improved tolerability and participation [36, 72]. This was particularly evident for children and people with learning disabilities, for whom environmental changes helped reduce anxiety and improve cooperation [36, 72]. Reported modifications included dimmed lighting, visual distractions such as slow-moving colour effects, use of rhythmic music, and tactile aids such as weighted wraps, creating a calming sensory experience [36]. Evidence for animal-assisted therapy remained inconclusive [55].

##### Literacy-sensitive education and technology-supported engagement

Educational and motivational interventions, especially those tailored to literacy level, supported with clear individualized materials or videos with explanations, and delivered through outreach (e.g., mobile clinics, dental camps) were widely recommended and described as acceptable and helpful [20, 34, 41, 52, 63]. Technological tools, including digital smile design, photographs, visual aids, teledentistry, and mobile-health applications, enhance understanding, set expectations, support decision-making, and assist with system navigation, thereby improving acceptability [21, 28]. These interventions also complemented shared decision-making by ensuring patients were informed and able to participate actively in care planning [19–21].

##### Culturally responsive and preference-sensitive care

Culturally competent and linguistically accessible care facilitated acceptability [20, 25, 60, 70, 73]. Interpreter services, culturally sensitive and safe practices, gender-sensitive approaches, and improved intercultural communication were reported to enhance trust and improve acceptability and use among diverse groups [20, 25, 32, 60, 68, 70, 73]. Respect for cultural values and openness to patient preferences, including preferences for minimally invasive, painless, drill-sparing, or shorter procedures, also facilitated acceptance [26, 46]. Procedure specific reviews highlighted acceptance of certain treatments such as SDF [27, 35, 47, 66] over general anesthesia; Hall Technique and atraumatic restorative technique [46, 61]; repair of partially defective restorations when longevity was reasonable [45]; and vacuum-formed retainers compared to Hawley retainers [51]. In prosthodontics, custom planning and anticipated functional benefits influenced choices, with implants or fixed partial dentures typically preferred over removable options due to enhanced mastication and improved appearance [23, 30].

##### Shared decision-making and supportive clinic environments

Trusting and continuous relationships with dental providers were central facilitators of acceptability [26, 47, 61, 72]. Empathetic, patient-centred communication; professional competence; and a user-friendly clinical environment consistently supported engagement [32, 58, 64, 73]. Shared decision-making, including the use of patient decision aids, clear explanation of procedures, and delivery of patient-centered care, was both desired and facilitated greater acceptance [19, 22, 54, 58, 72]. Support from a dedicated case manager, especially for pediatric patients, further enhanced continuity of care [44]. Patients valued providers with communication accommodations such as sign-language capability, expertise in special-care dentistry, consultant leadership for complex cases, and skill-mix approaches where hygienist-delivered preventive care was well-accepted [20, 29, 63, 69]. Clarity regarding roles within the dental team and explicit teaching of self-care skills further supported uptake [38, 63]. Adjustments in the waiting area, such as access to drinks, television, or picture-based reading materials and clear communication about appointment delays were also beneficial for reducing stress before treatment [72].

##### Practical and system-level support

Facilitators addressing practical constraints improved patients’ feasibility of attending and completing care. Reduced treatment costs, robust public dental benefits, especially for children with special healthcare needs, and effective system-level programs supported uptake [63, 66]. Flexible scheduling, efficient appointment systems, and enhanced continuity of care helped address competing obligations and reduced the likelihood of missed appointments [71, 73]. In emergency contexts, willingness to seek and accept care was higher than for routine dental visits, highlighting the importance of timely and responsive services [32, 37].

### Provider perspectives

#### Barriers to accepting patients

##### Gaps in clinical skills and procedural confidence

Providers reported insufficient training and skills, particularly in behaviour management and in caring for patients with complex medical and cognitive needs [20, 53, 54, 58, 71, 72]. These gaps contributed to uncertainty and reduced providers’ willingness to accept such patients into their practices [20, 53, 54, 58, 71, 72]. In addition, some providers reported limited comfort with certain procedures. For example, minimally invasive or conservative treatment approaches, although generally more acceptable to patients, may be less preferred by some providers when these techniques lack familiar tactile or sensory feedback [24]. Methods such as laser therapy or air-/sono-abrasion can make it difficult for clinicians to immediately assess the extent of caries removal, potentially reducing provider confidence and influencing treatment acceptance [24]. Similarly, with SDF, some providers express reservations related to anticipated esthetic outcomes and concerns about parental acceptance of the characteristic black staining, making them hesitant to offer it as a primary treatment option [47].

##### Technical and logistical difficulties

Ongoing technological advancements in dentistry, while improving the sophistication and capabilities of care, have also raised patients’ expectations [21, 28]. At the same time, they place additional demands on dental professionals, who must acquire new technical skills and become proficient with associated software and equipment [21, 28]. The learning curve for adopting these technologies varies among practitioners and often requires substantial additional training and practice [21, 28]. In addition, the need for specialized equipment or personnel, often involving significant financial investment, can deter providers from offering or accepting patients who require technologically advanced or resource-intensive treatments [21, 48]. Logistical demands such as longer appointment durations, extended procedural times, the need for specialized equipment, and additional trained staff posed significant barriers to accepting high-risk or medically complex patients [24, 48]. Virtual consultations for patients who cannot make an in-person appointment appear to offer an appropriate solution to accept patients and provide some level of care; however, uncertainties regarding teledentistry, including accuracy of assessments, quality of digital transmission, as well as equipment costs and privacy risks associated with digital systems, were also reported [28].

##### Communication, care planning, and capacity constraints

Providers identified difficulties in communication and shared decision-making as barriers to care [22]. Mismatched provider-patient priorities and differing expectations were sometimes viewed as requiring compromises clinicians did not prefer in their treatment planning and care delivery, restricting their willingness to accept some patients [22, 23]. Structural constraints, including limited clinical space, resource shortages, and insufficient administrative support disrupted care planning and restricted the full utilization of dental care professionals [63, 67]. Capacity limitations within teaching-hospital environments also shaped case acceptance patterns, with consultants prioritizing complex cases while routine cases were often accepted primarily for undergraduate or postgraduate training purposes [76].

##### System-level and sociodemographic barriers

Reported barriers included stigma and bias toward certain population groups, as well as assumptions about patients’ beliefs, behaviours, or likelihood of adherence, which in some cases led to under-recommending appropriate procedures [19, 20]. Providers also consistently cited financial barriers, such as inadequate or absent compensation, low or denied reimbursement, delayed payments, and generally weak remuneration structures as significant obstacles to delivering time-intensive or complex care for patients insured through publicly funded programs [20, 63, 66, 67, 72]. Administrative burden associated with these systems, coupled with high rates of missed appointments and last-minute cancellations, further undermined the feasibility of providing care to these populations [67]. Broader structural constraints within the system also limited providers’ ability to accept and manage patients. Weak pre-communication systems impeded information transfer and care coordination, especially for children with disabilities [20]. Limited interdisciplinary collaboration was also reported, with oral health frequently overlooked by other healthcare professionals, leading to missed opportunities for appropriate guidance and inadequate integration of dental considerations into broader care plans [33]. System-level assessments further described a lack of continuity of care and limited collaboration both within and between services when supporting people with disabilities, creating additional barriers to accessing appropriate dental care [71].

#### Facilitators to accepting patients

##### Efficient, adaptable, and patient-centered care approaches

Conservative procedures with shorter durations, lower technical demands, or minimal need for patient cooperation enhanced provider acceptability [35, 46, 61]. Techniques aligned with providers’ existing skills and confidence were viewed positively, particularly when time pressures were reduced [24, 46]. The use of sensory aids and sensory-adapted dental environments also eased patient management and supported successful treatment, especially for children or patients with behavioral or cognitive challenges [36, 72].

##### Financial and structural support

Adequate financial and policy frameworks facilitated providers’ willingness to deliver care, particularly for populations requiring more time or specialized management [66, 71]. For example, dental providers in British Columbia reported that reimbursement should reflect the increased time and responsibility associated with treating persons with special healthcare needs [66]. Clearly defined regulatory and remuneration systems that incentivized the use of skill-mix improved efficiency by enabling appropriate delegation of tasks [63, 76]. Team-based models and effective delegation improved productivity and operational efficiency within dental services, expanding capacity to manage needs-led care delivery [63, 69, 71, 76].

##### Care coordination and referral systems

Effective pre-visit communication and referral systems supported predictable, patient-centered care by improving information flow and coordination across services [20, 21, 76]. Receiving essential medical and behavioural information in advance enabled dental professionals to prepare and tailor treatment, particularly for children with disabilities [20]. Digital tools such as digital smile design facilitated treatment planning by allowing clinicians to simulate outcomes, analyze facial and dental proportions, and preview treatment options [21]. Clearly defined referral protocols improved coordination within specialist services, with both referring and receiving clinicians sharing responsibility for referral quality [76].

##### Training, mentorship, and workforce development

Targeted training opportunities, particularly in disability care, communication strategies, and behavioral management, were identified as key facilitators of provider confidence and preparedness [20, 22, 72]. Mentorship programs and partnerships with specialist teams strengthened providers’ ability to manage patients with complex or specialized needs [20, 22, 48, 72]. 

##### Communication and support tools

Digital visualization and planning tools, teledentistry for triage and communication, and dental mobile-health applications supported expectation setting, streamlined workflows, and individualized care [21, 23, 28, 53]. Passive immobilization tools supported providers in accepting patients with behavioural needs; however, for the usage of such tools, clear and explicit informed consent was considered crucial [37].

## Discussion

Acceptability is a critical yet often overlooked dimension of oral health care access, shaping both patients’ decisions to seek care and providers’ willingness to deliver it. As public funding for oral health care coverage expands in Canada, ensuring that care is not only affordable and available but also acceptable will be essential to achieving equitable uptake. This scoping review of reviews highlights the complex, multi-faceted nature of acceptability in oral health care, demonstrating how patient-, provider-, and system-level factors interact to influence access and utilization. While financial and structural barriers have traditionally been the focus of policy interventions, this review attempts to comprehensively assess all dimensions to equitably improve acceptability of oral health care services.

### Contextual and intersecting influences

Our findings indicate that acceptability is not static, but highly context-dependent, shaped by the interaction of patient identity, health system characteristics, and the circumstances surrounding care. In emergency situations, for example, patients may be more willing to accept treatments they would otherwise refuse, prioritizing immediacy or necessity over routine care [32, 37]. Cultural norms and place of residence further influence perceptions of which treatments are appropriate, with some populations being more open to certain procedures than others [33, 60, 62, 77].

Providers, in turn, operate within health systems that shape what care can realistically be offered. Models of care vary across jurisdictions, meaning that practices considered acceptable in one context may not translate directly to another. Even within individual dental settings, patients and providers may prioritize different outcomes. Patients may place greater emphasis on esthetics, while providers may prioritize durability, efficiency, or clinical effectiveness, leading to mismatched expectations [22, 35, 45–47, 51, 61]. Similarly, innovations such as teledentistry or minimally invasive treatments may function as facilitators or barriers depending on whether patients value convenience, comfort, esthetics, or perceived effectiveness [21, 24, 26, 28, 53]. Collectively, these findings underscore that acceptability is best understood as a dynamic process of negotiating trade-offs among competing priorities, shaped by patient expectations, provider practices, and the broader systems that structure their interactions.

### Relationship to broader healthcare literature

Although most included reviews did not explicitly define or measure “acceptability,” their findings aligned with the TFA and echoed patterns in the broader healthcare literature [9, 10]. Core domains such as affective attitude (emotional response to care), burden (perceived effort required), ethicality (alignment with personal values), and self-efficacy (confidence engaging with care) were consistently central to care uptake [9, 10]. Simultaneously, oral health exhibited distinct characteristics that set it apart from other health contexts. Factors such as fear rooted in childhood dental experiences, esthetic concerns related to visible treatments, and aversions to procedures such as SDF emerged as unique determinants operating alongside more general considerations of access to or quality of care [19, 23, 26, 27, 32, 35, 42, 47, 50, 58, 61, 66, 68, 71, 73]. These findings suggest that general acceptability frameworks require contextual adaptation when applied to oral health care.

Additionally, the dimensions of access described by Levesque et al. [8] are not mutually exclusive, and some themes identified in this review, such as cost and logistical concerns, intersect with affordability, availability, or appropriateness. This review examined these factors specifically in terms of how they shape patients’ and providers’ willingness to seek, deliver, or engage with care, which is the defining function of acceptability within the access framework [6].

### Policy implications

Building on these findings, we developed a tailored framework for oral health care acceptability intended as a practical tool for policy actors to use in the design and evaluation of dental programs (Fig. 2). At the patient level, interventions should integrate behavioral, educational, and culturally responsive strategies to improve trust, understanding, and comfort [20, 30–32, 34, 37, 44, 47, 52, 56, 60, 67, 71]. At the provider level, workforce development, mentorship, skill-mix optimization, and appropriate financial incentives are critical to enabling the delivery of acceptable care to diverse and complex populations [20, 63, 65, 66, 69, 71, 72]. At the system level, coordination mechanisms, including clear referral pathways, digital supports, and interprofessional collaboration can help bridge gaps between patient needs and provider capacity [20, 21, 28, 53].

The interaction between patient and provider acceptability highlights the need for a dual focus in access-oriented policy: enhancing patients’ readiness and willingness to engage with care while simultaneously supporting providers to deliver care that is both clinically appropriate and acceptable. This approach is essentially “reorienting health services”, one of the five dimensions identified in the Ottawa Charter, making health services more accessible to a larger population [84].

These implications are especially relevant in the Canadian context, where the CDCP has been implemented to reduce financial barriers to dental care for eligible residents. Applying an acceptability lens to program eligibility criteria, covered services, and provider remuneration structures may improve service utilization and ensure that publicly funded dental programs are not only accessible, but also meaningful and equitable.

### Strengths and limitations

This review has several strengths. It draws on a broad body of peer-reviewed and grey literature spanning multiple populations, dental settings, and procedural contexts. The use of abductive thematic analysis enabled an in-depth synthesis that integrated inductive insights from the data with established theoretical frameworks, strengthening the interpretive rigor of the findings. Notably, many included reviews did not frame their findings as acceptability; our inclusion of proxy factors, combined with the team's clinical background and theoretical lens, surfaced evidence the original authors had not labelled as such. To our knowledge, this is also among the first reviews to apply an acceptability lens specifically to oral health care and to synthesize patient and provider perspectives within a single framework.

Several limitations should also be noted. The interpretive latitude described above is also a limitation. As a scoping review of reviews, this synthesis involved interpretation at multiple levels. The included reviews reflected the conceptual emphases and reporting practices of their original authors, and our team applied an additional layer of interpretation when classifying and synthesizing findings across sources. While necessary for mapping the evidence, this process increases the potential for subjectivity in how acceptability was represented, and others applying a different lens might have framed some findings differently. Conceptual variability across reviews, reliance on proxy constructs such as satisfaction or preference, and the absence of a formal critical appraisal introduce further uncertainty regarding the robustness of the evidence base. Additionally, overlapping primary studies across reviews may have led to the overrepresentation of certain barriers or facilitators. 

A further limitation concerns the uneven distribution of evidence across perspectives. Provider-reported determinants were drawn from substantially fewer reviews than patient-reported ones, and several provider-side themes rested on a small number of sources. This imbalance means the provider dimension of the framework is supported by a thinner evidence base and should be interpreted with greater caution. It may reflect both a broader tendency in the literature to foreground patient over provider perspectives on acceptability and the limited sensitivity of our search and review-of-reviews design to provider-focused sources. Restricting inclusion to English- and French-language literature may have excluded relevant evidence and perspectives, which is a notable consideration given that acceptability is culturally shaped. In addition, grey literature was identified through ranked web and custom search-engine results rather than systematic database searching, so some relevant sources may have been missed and this component is less readily reproducible. As searches were conducted at a single time point in mid-2025, the findings also represent a snapshot of an evolving evidence and policy landscape. 

Although the inclusion of studies from multiple countries broadened the scope of evidence, differences in health system structures and unique cultural factors across the populations represented in the included reviews may limit comparability and generalizability across contexts. Finally, because acceptability is dynamic and condition-specific, the proposed framework may not capture all contextual nuances. Nonetheless, it provides a valuable foundational structure for understanding acceptability in oral health care and highlights the need for future research that explicitly defines and measures this construct.

## Conclusions

This scoping review demonstrates that acceptability is a critical, context-dependent dimension of access to oral health care, shaped jointly by patient experiences and provider practices. Emotional, cultural, procedural, technical, and system-level factors interact to influence whether oral health services are ultimately utilized. Integrating acceptability into oral health policy and program design is essential for addressing persistent gaps between coverage, access, and utilization, and for ensuring that care is not only affordable and available, but genuinely acceptable to those who seek and deliver it.

## Supplementary Information

Additional file 1: PRISMA Checklist [17]. Additional File 1 contains a table that lists the items for the Preferred Reporting Items for Systematic reviews and Meta-Analyses extension for Scoping Reviews (PRISMA-ScR) Checklist and describes how these items were addressed in the manuscript.

Additional file 2: Sample Indexed Database Search Query. Additional File 2 contains a table that lists the Ovid MEDLINE search query, adapted for the other indexed databases and grey literature.

## Data Availability

The data generated and analyzed during this review are available from the corresponding author on reasonable request.

## Abbreviations

CDCP: Canadian Dental Care Plan
TFA: Theoretical Framework of Acceptability
UK: United Kingdom
PRISMA-ScR: Preferred Reporting Items for Systematic reviews and Meta-Analyses extension for Scoping Reviews
2SLGBTQ+: Two-Spirit, Lesbian, Gay, Bisexual, Transgender, and Queer
SDF: Silver Diamine Fluoride
