# Supplementary material for: Acceptability of oral health care: a scoping review of reviews to inform framework development
Source: BMC Oral Health. 2026 Jul 31;26:1830. doi: 10.1186/s12903-026-09124-6 (PMC13616125; doi:10.1186/s12903-026-09124-6)
Supplement: Supplementary file 1 [file 12903_2026_9124_MOESM1_ESM.docx]

**Additional File 1: PRISMA Checklist (11)**

| **Section** | **Item** | **PRISMA-ScR Checklist Item** | **Area Reported** |
| --- | --- | --- | --- |
| **Title** | | | |
| Title | 1 | Identify the report as a scoping review. | Scoping review noted in the title. |
| **Abstract** | | | |
| Structured summary | 2 | Provide a structured summary that includes (as applicable): background, objectives, eligibility criteria, sources of evidence, charting methods, results, and conclusions that relate to the review questions and objectives. | Structured summary items included in abstract. |
| **Introduction** | | | |
| Rationale | 3 | Describe the rationale for the review in the context of what is already known. Explain why the review questions/objectives lend themselves to a scoping review approach. | Rationale described throughout the Introduction. |
| Objectives | 4 | Provide an explicit statement of the questions and objectives being addressed with reference to their key elements (e.g., population or participants, concepts, and context) or other relevant key elements used to conceptualize the review questions and/or objectives. | Question of interest guiding the review included in paragraph 4 in the Introduction. |
| **Methods** | | | |
| Protocol and registration | 5 | Indicate whether a review protocol exists; state if and where it can be accessed (e.g., a Web address); and if available, provide registration information, including the registration number. | Review protocol was not posted. |
| Eligibility criteria | 6 | Specify characteristics of the sources of evidence used as eligibility criteria (e.g., years considered, language, and publication status), and provide a rationale. | Characteristics described Search Strategy and Information Sources (Methods). |
| Information sources* | 7 | Describe all information sources in the search (e.g., databases with dates of coverage and contact with authors to identify additional sources), as well as the date the most recent search was executed. | Sources described Search Strategy and Information Sources (Methods). |
| Search | 8 | Present the full electronic search strategy for at least 1 database, including any limits used, such that it could be repeated. | Sample search strategy included in Additional File 2. |
| Selection of sources of evidence† | 9 | State the process for selecting sources of evidence (i.e., screening and eligibility) included in the scoping review. | Inclusion and exclusion criteria described in Table 1. |
| Data charting process‡ | 10 | Describe the methods of charting data from the included sources of evidence (e.g., calibrated forms or forms that have been tested by the team before their use, and whether data charting was done independently or in duplicate) and any processes for obtaining and confirming data from investigators. | Methods for how information from the included studies were described, extracted and analyzed are described under Data Extraction and Analysis (Methods). |
| Data items | 11 | List and define all variables for which data were sought and any assumptions and simplifications made. | Items extracted are described under Data Extraction and Analysis (Methods). |
| Critical appraisal of individual sources of evidence§ | 12 | If done, provide a rationale for conducting a critical appraisal of included sources of evidence; describe the methods used and how this information was used in any data synthesis (if appropriate). | Critical appraisal was not conducted. |
| Synthesis of results | 13 | Describe the methods of handling and summarizing the data that were charted. | Thematic analysis approach described under Data Extraction and Analysis (Methods). |
| **Results** | | | |
| Selection of sources of evidence | 14 | Give numbers of sources of evidence screened, assessed for eligibility, and included in the review, with reasons for exclusions at each stage, ideally using a flow diagram. | Flow diagram included as Figure 1. |
| Characteristics of sources of evidence | 15 | For each source of evidence, present characteristics for which data were charted and provide the citations. | Data items extracted are presented in Table 2. |
| Critical appraisal within sources of evidence | 16 | If done, present data on critical appraisal of included sources of evidence (see item 12). | Critical appraisal was not conducted. |
| Results of individual sources of evidence | 17 | For each included source of evidence, present the relevant data that were charted that relate to the review questions and objectives. | Outcomes across included studies are synthesized in text form as part of the thematic analysis. |
| Synthesis of results | 18 | Summarize and/or present the charting results as they relate to the review questions and objectives. | Outcomes across included studies are synthesized in text form as part of the thematic analysis. |
| **Discussion** | | | |
| Summary of evidence | 19 | Summarize the main results (including an overview of concepts, themes, and types of evidence available), link to the review questions and objectives, and consider the relevance to key groups. | Overview of themes are included in Figure 2. Results are discussed in terms of relevance throughout the Discussion. |
| Limitations | 20 | Discuss the limitations of the scoping review process. | Limitations of the scoping review process are described under Strengths and limitations (Discussion). |
| Conclusions | 21 | Provide a general interpretation of the results with respect to the review questions and objectives, as well as potential implications and/or next steps. | Conclusions are described under Conclusions. |
| **Funding** | | | |
| Funding | 22 | Describe sources of funding for the included sources of evidence, as well as sources of funding for the scoping review. Describe the role of the funders of the scoping review. | Details reported under Funding. |

This table lists the items for the Preferred Reporting Items for Systematic reviews and Meta-Analyses extension for Scoping Reviews (PRISMA-ScR) Checklist and describes how these items were addressed in the manuscript.
