# Supplementary material for: Acceptability of oral health care: a scoping review of reviews to inform framework development
Source: BMC Oral Health. 2026 Jul 31;26:1830. doi: 10.1186/s12903-026-09124-6 (PMC13616125; doi:10.1186/s12903-026-09124-6)
Supplement: Supplementary file 2 [file 12903_2026_9124_MOESM2_ESM.docx]

**Additional File 2: Sample Indexed Database Search Query**

| **#** | **Searches (ALL <1946 to June 06, 2025>)** | **Results** |
| --- | --- | --- |
| 1 | Oral Health/ or Community Dentistry/ or Comprehensive Dental Care/ or exp Dental Care/ or Dental Caries/pc, th or Dental Health Services/ or Dental Prophylaxis/ or Dental Restoration, Permanent/ or Dental Restoration, Temporary/ or Dental Scaling/ or Dentistry/ or General Practice, Dental/ or Geriatric Dentistry/ or Pediatric Dentistry/ or Periodontics/ or Preventive Dentistry/ or Public Health Dentistry/ or Radiography, Dental/ or exp Root Canal Therapy/ or School Dentistry/ or State Dentistry/ or exp Tooth Extraction/ or Crowns/ | 210385 |
| 2 | (dental or dentist* or "oral health*" or "oral care" or "oral status" or periodont*).ti. | 258921 |
| 3 | ("dental care" or "oral health care" or ((dental or dentist* or oral or periodont*) adj3 (care or service* or visit* or treat*)) or ((prevent* or prophyla* or "public health" or community) adj3 dent*) or (("root canal*" or "dental pulp") adj3 (treat* or therap*)) or ((dental or dentist* or tooth or teeth*) adj5 (clean* or checkup* or "check up*" or exam* or extract* or filling* or radiograph* or restor* or scaling or visit* or xray* or "x-ray*")) or ((caries or cavity or cavities) adj3 (prevent* or care or treat*)) or (extract* adj3 (tooth or teeth)) or (fluorid* adj3 treat*) or ((dentist* or dental* or "oral health") adj3 appointment*) or periodontics or dentistry).kf,kw,ti. | 103303 |
| 4 | (dental or dentist* or "oral health*" or "oral care" or "oral status" or periodont* or tooth or teeth).ab. and (Utilization Review/ or "Facilities and Services Utilization"/ or Treatment Refusal/ or Refusal to Participate/ or "Treatment Adherence and Compliance"/ or Patient Compliance/ or No-Show Patients/ or Patient Dropouts/ or Patient Participation/ or Undertreatment/ or undertreat*.ti. or ((utilis* or utiliz* or uptak* or "use" or accessed or declin* or postpon* or delay* or avoid* or refuse? or refusing or refusal or discontinu* or dropout* or "drop* out*") adj3 (care or service* or treat* or therap* or visit* or appointment*)).ti. or ((patient or client*) adj3 (participat* or adher* or complian* or comply or complies or complied)).ti.) | 3065 |
| 5 | (attend* adj3 (clinic? or office?)).ti. and (dental or dentist* or "oral health*" or "oral care" or "oral status" or periodont* or tooth or teeth).ab. | 94 |
| 6 | (dental or dentist* or "oral health*" or "oral care" or "oral status" or periodont* or tooth or teeth).ab. and ((restor* or prevent* or routine) adj3 (care or treat* or service)).ti. | 1822 |
| 7 | 1 or 2 or 3 or 4 or 5 or 6 | 405042 |
| 8 | Patient Acceptance of Health Care/eh, px | 15043 |
| 9 | *Patient Acceptance of Health Care/ | 34415 |
| 10 | Patient Acceptance of Health Care/ and (accept* or unaccept* or appropriate* or inappropriate* or tolerab* or intolerab* or "object* to" or objected or objection*).ab,kf,kw,ti. | 27726 |
| 11 | ((accept* or unacceptab* or appropriate* or inapprop* or tolerab* or intolerab* or "object* to" or objected or objection*) and (care or service* or treat* or therap* or fluorid*)).kf,kw,ti. | 26088 |
| 12 | (barrier* or facilitator* or ((predispos* or enabl* or need*) adj3 factor*) or predictor*).kf,kw,ti. and (acceptability or acceptable or acceptance).ab,kf,kw,ti. | 4638 |
| 13 | 8 or 9 or 10 or 11 or 12 | 76110 |
| 14 | 7 and 13 | 1420 |
| 15 | Utilization Review/ or "Facilities and Services Utilization"/ or Treatment Refusal/ or Refusal to Participate/ or "Treatment Adherence and Compliance"/ or Patient Compliance/ or No-Show Patients/ or Patient Dropouts/ or Patient Participation/ or Undertreatment/ or undertreat*.kf,kw,ti. or ((utilis* or utiliz* or uptak* or "use" or accessed or declin* or postpon* or delay* or avoid* or refuse? or refusing or refusal or discontinu* or dropout* or "drop* out*") adj3 (care or service* or treat* or therap* or visit* or appointment*)).kf,kw,ti. or ((patient or client*) adj3 (participat* or adher* or complian* or comply or complies or complied)).kf,kw,ti. [SERVICE UTILIZATION] | 237902 |
| 16 | Health Knowledge, Attitudes, Practice/ or Attitude to Health/ or Attitude/ or Emotions/ or Affect/ or Anxiety/ or Dental Anxiety/ or Fear/ or Patient Satisfaction/ or Patient Preference/ or (attitud* or opinion* or emotion* or feel* or fear* or anxiet* or anxious* or satisf* or prefer* or "user experience*" or "patient experience*").kf,kw,ti. [ATTITUDES/EMOTIONS] | 886586 |
| 17 | (convenien* or inconvenien* or ease or easy or easily or burden* or hassle*).kf,kw,ti. [BURDEN] | 85546 |
| 18 | Perception/ or exp Health Belief Model/ or px.fs. or (perception or perceiv* or expectation* or belief* or thoughts or think*).kf,kw,ti. [PERCEIVED EFFECTIVENESS] | 1469363 |
| 19 | Trust/ or Respect/ or Ethics/ or Ethics, Professional/ or Ethics, Clinical/ or Ethics, Dental/ or Morals/ or Stigma/ or (ethic* or moral* or trust* or distrust* or mistrust* or respect* or disrespect* or "supportive care" or "unsupportive care" or stigma* or welcome* or unwelcome*).kf,kw,ti. [ETHICALITY] | 189284 |
| 20 | Comprehension/ or Sense of Coherence/ or (coheren* or understand* or aware or awareness or comprehend*).kf,kw,ti. [INTERVENTION COHERENCE] | 211839 |
| 21 | ("opportunity cost*" or willingness).kf,kw,ti. [OPPORTUNITY COSTS] | 8605 |
| 22 | Self-Efficacy/ or Self Concept/ or ("self efficacy" or "self confidence" or "self concept").kf,kw,ti. [SELF-EFFICACY] | 80448 |
| 23 | Culture/ or Cultural Characteristics/ or Culturally Competent Care/ or Cultural Competency/ or Cross-Cultural Comparison/ or Social Values/ or eh.fs. or (cultur* or values).kf,kw,ti. [CULTURAL FACTORS] | 595861 |
| 24 | 16 or 17 or 18 or 19 or 20 or 21 or 22 or 23 | 2890689 |
| 25 | 7 and 15 and 24 | 1363 |
| 26 | 14 or 25 | 2670 |
| 27 | (Review or "Systematic Review" or "Meta Analysis").pt. or (review.ti. not medline.st.) or ("meta-analy*" or metaanaly* or "meta analy*" or metanaly* or (pooled adj3 study) or (pooled adj3 analysis) or ((literature or systematic or evidence or quantitative or qualitative or "mixed methods" or integrative or research or critical or studies or umbrella or scoping or realist or rapid or narrative) adj5 (synthesis or review or overview)) or "review of reviews" or "knowledge synthesis").ti. or ("in this review".ab. not medline.st.) or "Cochrane Database Syst Rev".ja. or "Syst Rev".ja. [REVIEWS] | 3920091 |
| 28 | 26 and 27 | 178 |
| 29 | limit 28 to (english or french) | 172 |

This table lists the Ovid MEDLINE search query, adapted for the other indexed databases and grey literature.
